# Diagnostic Utility of Separate Anti-Ro60 and Anti-Ro52/TRIM21 Antibody Detection in Autoimmune Diseases

**DOI:** 10.3389/fimmu.2019.00444

**Published:** 2019-03-12

**Authors:** Ailsa Robbins, Maxime Hentzien, Segolene Toquet, Kevin Didier, Amelie Servettaz, Bach-Nga Pham, Delphine Giusti

**Affiliations:** ^1^Department of Internal Medicine, Infectious Diseases, and Clinical Immunology, Robert Debré Hospital, Reims University Hospitals, Reims, France; ^2^Department of Internal Medicine, Robert Debré Hospital, Reims University Hospitals, Reims, France; ^3^Laboratory of Dermatology, Faculty of Medicine, University of Reims Champagne-Ardenne, Reims, France; ^4^Laboratory of Immunology, Reims University Hospital, University of Reims Champagne-Ardenne, Reims, France

**Keywords:** TRIM21, anti-Ro52 antibodies, anti-Ro60 antibodies, anti-SSA antibodies, autoimmune diseases, connective tissue disease, primary Sjögren's syndrome, systemic lupus

## Abstract

Anti-SS-A antibodies are often sought for in autoimmune diseases diagnosis. Two different target proteins have actually been identified: Ro52 and Ro60. Clinical and immunological associations seem different depending on anti-Ro52 or anti-Ro60 antibodies presence. However, due to a heterogeneous presentation in the literature, some immunology laboratories in France have stopped providing anti-Ro52 antibody findings. We report here a new hospital study designed to determine the diagnostic utility of the separate detection of anti-Ro52 and anti-Ro60 antibodies. We conducted a retrospective, observational study, including every adult patient with positive antinuclear antibodies (ANA) tested in our immunology laboratory, and associated with anti-Ro52 and/or anti-Ro60 antibodies, between 2011 and 2014. Out of 13032 sera tested for ANA, 399 adults had antibodies to Ro52 and/or Ro60; 81.7% were female, with a mean age of 54.5 ± 17.0 years. Anti-Ro52 antibodies were found in 75.7% of the patients and anti-Ro60 antibodies in 56.9%. Among them, 43.1% were classified in the Ro52 + Ro60- group, 32.6% in the Ro52 + Ro60 + group and 24.3% in the Ro52-Ro60+ group. In the Ro52-Ro60+ group, systemic lupus was the most frequent diagnosis (48.5%), with a possible association with antiphospholipid antibodies (anti-cardiolipin antibodies: OR 2.5 (CI95 [1.0–5.0], *p* = 0.05) and lupus anticoagulant {OR 3.6 (CI95 [1.10–10.0] *p* = 0.02)}. In the Ro52+Ro60+, primary Sjögren Syndrome was the most likely (OR 4.2 95% CI [2.1–8.3] *p* < 10^−4^), especially in patients Ro52+Ro60+La+. Patients with isolated anti-Ro52 had a wider variety of diseases associated, but among auto-immune diseases they were more prone to inflammatory myositis (OR 10.5 [1.4–81.7], *p* = 0.02) and inflammatory rheumatism (OR 4.6 [1.6–13.8], *p* = 0.006) in contrast to systemic lupus (OR 0.2 [0.1–0.3], *p* < 10^−4^) or primary Sjögren's syndrome (OR 0.1 [0.06–0.2], *p* < 10^−4^). We therefore suggest that, when anti-ENA antibodies are prescribed, it should include separate anti-Ro52 and anti-Ro60 antibodies determination. To go even further, we would like to suggest a change in ENA nomenclature to avoid confusion, abandoning the anti-SS-A label in favor of the anti-Ro52/TRIM21 or anti-Ro60 antibody for a clearer designation.

## Introduction

Antibodies to the Ro/SS-A system are classically described in association with autoimmune diseases (AID) such as systemic lupus, and Sjögren's syndrome (1). More recently, two different target proteins have been differentiated: Ro60 (60 kDa) and Ro52 (52 kDa), that have distinct biochemical and immunological functions (2, 3). Indeed, Ro52 corresponds to TRIM21, belonging to the Tripartite Motif Protein (TRIM) family (4). It is implicated in protein ubiquitination, pro-inflammatory states (interleukin 2) and apoptosis mechanisms (4–7). Ro60 is a protein component of small cytoplasmic ribonucleoprotein complexes (hY-RNA complexes) which can bind misfolded, non-coding RNA, probably taking part in their final degradation. It also seems to have an important function in cell survival after ultraviolet irradiation (8).

Clinically, it has been suggested that different associations of anti-Ro60 (Ro60 Ab) or anti-Ro52 antibodies (Ro52 Ab) in patients may associate with different phenotypes such as systemic lupus, neonatal lupus, and fetal atrioventricular blockade, primary Sjögren's syndrome, or inflammatory myositis (9–24). However, some authors question the diagnostic utility of individual detection of Ro52 and Ro60 Ab (17, 25) as their association in certain studies concerning the Ro-SS-A system are contradictory. This is the case for systemic lupus for example (15, 17). Discrepancies are also found about the association with primary Sjögren's syndrome: studies based on patients with Sjögren's syndrome find a strong association with Ro52 Ab (26, 27), whereas studies based on Ro52 Ab positive sera are more heterogeneous, some showing an association with this disease (3, 12, 16, 18) and some showing a less frequent association than with Ro60 Ab (15). Therefore, some authors suggest that detection of Ro60 Ab alone is sufficient for AID diagnosis. Based on these heterogeneous data, some immunology laboratories in France no longer test for Ro52 Ab or, at least, do not report the results of its detection. This can be confusing for clinicians as they often do not clearly know what has been tested for, when receiving an “anti-SS-A antibodies” result (reactivity to Ro60, Ro52 or a mixture of both). Only a few studies explicitly looked for differences between patients with isolated Ro52 Ab, isolated Ro60 Ab and double positive patients (15, 16, 18).

This study was therefore conducted to better characterize the possible different phenotypes emerging when Ro52 Ab and Ro60 Ab are analyzed separately, and thus, to establish the relevance of their separate detection.

## Patients and Methods

We performed a retrospective review of every serum from patients tested for anti-nuclear antibodies (ANA) by the immunology laboratory at Reims University hospital, from January 2011 to November 2014. Among patients with positive ANA, we included every adult (≥ 18-year-old) hospital-patient (hospitalized or consulting for any reason) with Ro52 and/or Ro60 Ab. Of note, our center has a 2,635 bed capacity, allowing around 99.600 in-patients and 319.700 out-patients a year, in various medical (immunology, hematology, oncology, neurology, gastroenterology, nephrology, respiratory diseases, etc.) and surgical wards.

In addition, 51 healthy persons from the blood and tissue donors bank were included as control group and were tested for anti-ENA antibodies.

## ANA Detection

ANA presence was tested by indirect immunofluorescence (IIF) on Hep-2000 cells-coated slides (Eurobio Ingen, Les Ulis, France). Slides were incubated with sequential dilutions of serum from the initial dilution of 1/100 to 1/1600 and revealed with FITC-bound anti-human IgG antibodies. The positivity threshold was set at a titer of 1/100. When ANA detection was positive, sera were screened for anti-Extractable Nuclear Antigens (ENA) antibodies.

## Anti- ENA Antibodies Quantitative Determination

Anti-ENA antibodies (including Ro52 and Ro60 Ab) were detected with multiplex fluorescent microsphere immunodetection (FIDIS Connective Profile MX117™, Theradiag®, Marne-La-Vallée, France) according to manufacturer's instructions. Briefly, uniformly sized microspheres of different colors (red to infrared) with bound antigens were used and analyzed simultaneously. The samples were diluted and mixed with the microspheres, and specific antibodies, if present, bound to the coated antigens and were revealed using a phycoerythrin conjugate in flow cytometry. Each analyte was identified using 2 lasers, allowing the identification of the colored signature of each bead and quantifying the ratio of analytes bound to it. The positivity threshold for all antibodies was set by the manufacturer at 40 arbitrary units (AU)/mL. The “ENA” microsphere mixture comprises recombinant antigens, SSA Ro52, SSB, centromere B (CENPB), Jo1, PmScl, PCNA and native purified antigens SSA Ro60, Scl70, SmRNP, Sm, U1RNP, ribosome, and Histone.

## Data Collection

We collected demographical, clinical and biological data at the time of ENA analysis from each medical record. The main data collected were vital status at last follow-up, presence of anemia (hemoglobin <130 g/L for men and <120 g/L for women), thrombopenia (platelet count <150 G/L), lymphopenia (lymphocyte count <1.5 G/L), hypergammaglobulinemia (gammaglobulin>15 g/L on plasmatic protein electrophoresis), renal insufficiency (glomerular filtration rate <60 mL/min/1.73 m^2^) and the number of lines of treatment. For patients with systemic sclerosis we also collected diagnosis of heart (pulmonary hypertension) or pulmonary (fibrosis) involvement. Concerning diagnosis, we chose to register the diagnosis as established by the referring physician at last follow-up. Indeed, auto-immune diseases diagnoses are often made by experienced clinicians, based on a body of evidence, not necessarily corresponding to international classification criteria.

Anti-ENA antibody specificities with the FIDIS™ technique were noted, as well as anti-dsDNA antibodies with a Radio-Immuno Assay Farr technique (positivity threshold ≥ 5UI) (Siemens Healthcare Diagnostics, Saint-Denis, France), anti-cyclic citrullinated peptide antibodies (anti-CCP) (ELISA, Immunoscan CCPlus™, Euro Diagnostica, Malmö, Sweden), Rheumatoid Factor (RF) (FIDIS Rheuma™, Theradiag®, Marne-La-Vallée, France), anti-thyroglobulin (TG) and anti-thyroperoxydase antibodies (TPO) (FIDIS Thyroid™, Theradiag®, Marne-La-Vallée, France), anti-β2GP1 and anti-cardiolipin antibodies (Varelisa™, Thermofisher Phadia, Uppsala, Sweden) and lupus anticoagulant (PTT-LA and RVV-Screen, Diagnostica Stago, Asnières, France) were sought for at any point during follow-up.

## Statistical analysis

Qualitative data were expressed in numbers and percentage. They were compared with a chi square test when possible, or with Fisher's exact test. Quantitative data were expressed in mean with their standard deviation and compared with Student's *t*-test, Kruskal-Wallis or Mann-Whitney test when appropriate. Logistic regression was used to calculate odds ratio with their confidence interval (SAS® 9.3 software). *p* < 0.05 was considered statistically significant.

## Results

During a 4 year-period, 7,821 out of 13,032 (60.0%) tested sera were positive for ANA detection. Among them, 399 patients were positive for Ro60 and/or Ro52 Ab as detected by the FIDIS™ technology. Conversely, none of the individuals from the control group (28) were found to have either Ro52 or Ro60 Ab.

**Figure 1:**
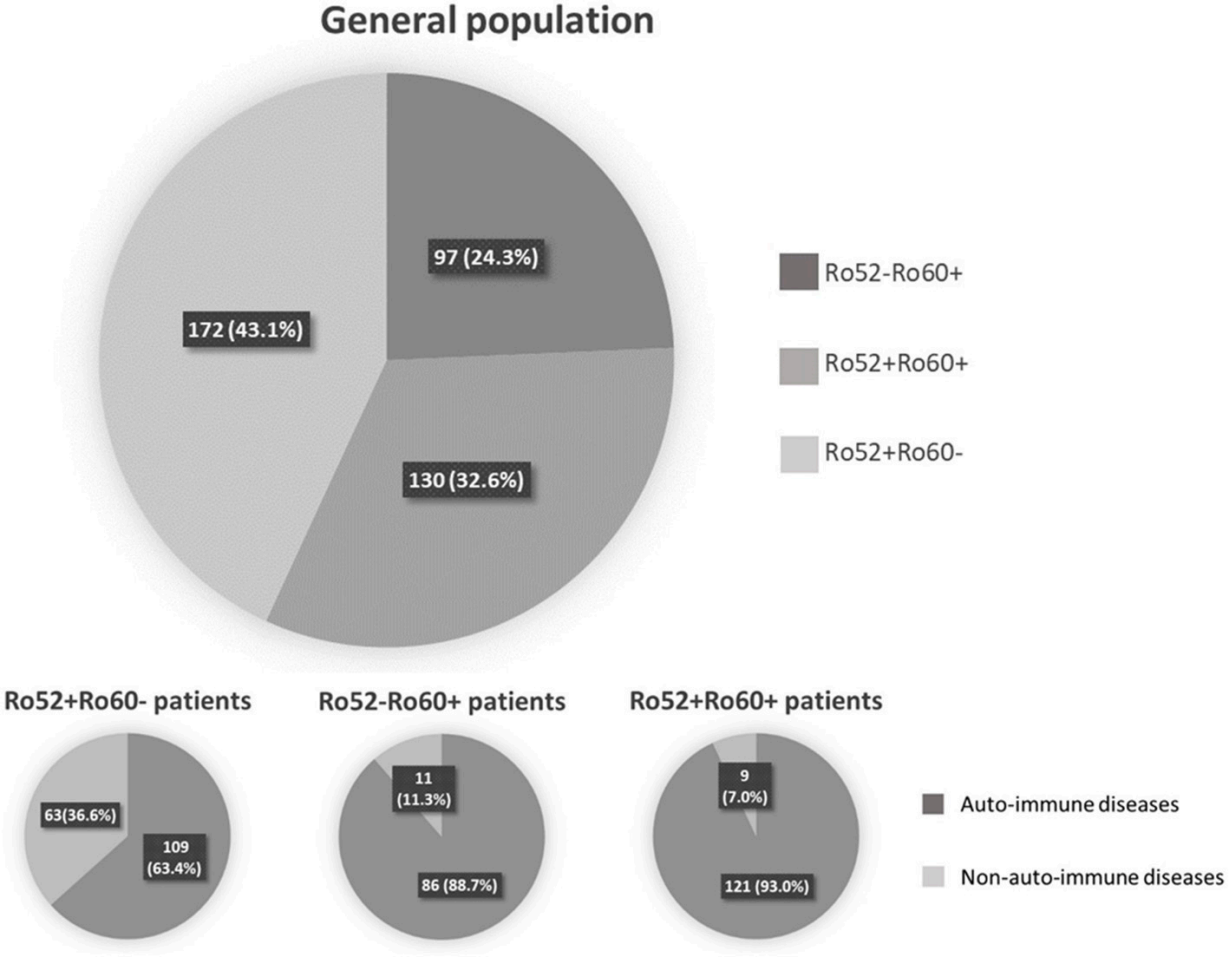
Repartition of patient depending on anti-Ro52 and/or anti-Ro60 antibodies presence or absence.

Among these 399 positive patients, 326 (81.7%) were female and mean age was 54.5 ± 17.0 years-old (range 18–92 years-old). An AID was diagnosed in 316 (79.2%) patients, whereas 70 (17.5%) had a non-autoimmune disease. Thirteen (3.3%) patients could not be classified because of missing data (6 patients) or because the referring physician did not conclude at last follow-up (7 patients) (Figure 1). The majority of the patients were followed in an Internal Medicine or Rheumatology ward (71.2%). Median follow-up was 3.0 years 95%IC [1.0–9.0], with a maximum of 33 years follow-up. Out of the 399 patients, 302 (75.7%) had Ro52 Ab and 227 (56.9%) had Ro60 Ab. There were 172 patients (43.1%) with isolated Ro52 Ab (Ro52+Ro60-), 97 (24.3%) with isolated Ro60 Ab (Ro52-Ro60+) and 130 (32.6%) double positive patients (Ro52+Ro60+) (Figure 1).

Patients in these three groups were different with respect to demographical, clinical and immunological data. Patients in the Ro52+Ro60- group were older (59.3 ± 17.8 vs. 51.6 ± 16.2 in the Ro52+Ro60+ group and 49.8 ± 14.4 in the Ro52-Ro60+ group, *p* < 10^−4^) and the female predominance was less marked (71.5% vs. 90.0% and 88.7% in the Ro52+Ro60+ and Ro52-Ro60+groups, respectively, *p* < 10^−4^). AID were the most represented pathologies in every group, even if this predominance was less striking in the Ro52+Ro60- group (*p* < 10^−4^) (Table 1). ANA median titer in IIF was the highest in the Ro52+Ro60+ group (*p* < 10^−4^) (Table 2).

**Table 1:** Clinical associations in the Ro52+Ro60-, Ro52+Ro60+ and Ro52-Ro60+ groups.

	**Antibody profiles**									
	**Ro52 + Ro60- (*****n*** **= 172)**			**Ro52 + Ro60 + (*****n*** **= 130)**			**Ro52 - Ro60 + (*****n*** **= 97)**			
	***n***	**% in the disease group**	**% in the Ab group**	***n***	**% in the disease group**	**% in the Ab group**	***n***	**% in the disease group**	**% in the Ab group**	p^*^
**Auto-Immune diseases (*****n*** **= 316)**	**109**	**34.5**	**63.4**	**121**	**38.3**	**93**	**86**	**27.2**	**88.7**	**<10**^**−4**^
Systemic Lupus (*n* = 122)	21	17.2	12.2	54	44.3	41.5	47	38.5	48.5	**<10**^**−4**^
Sjögren disease (*n* = 76)	12	15.8	7.0	51	67.1	39.2	13	17.1	13.4	**<10**^**−4**^
Systemic sclerosis (*n* = 12)	7	58.3	4.1	3	25.0	2.3	2	16.7	2.1	0.6
Inflammatory myositis (*n* = 18)	13	72.2	7.6	1	5.6	0.8	4	22.2	4.1	**0.01**
Inflammatory Rhumatism (*n* = 36)	22	61.1	12.8	4	11.1	3.1	10	27.8	10.3	0.01
Other auto-immune diseases (*n* = 52)	34	65.4	19.8	8	15.4	6.2	10	19.2	10.3	**0.01**
Malignancies (*n* = 17)	**12**	**70.6**	**7.0**	**3**	**17.6**	**2.3**	**2**	**11.8**	**2.1**	0.08
Infectious diseases (*n* = 15)	**11**	**73.3**	**6.4**	**3**	**20.0**	**2.3**	**1**	**6.7**	**1.0**	0.06
Other (*n* = 38)	**31**	**81.6**	**18.0**	**1**	**2.6**	**0.8**	**6**	**15.8**	**6.2**	**<10**^**−4**^
Not classified (*n* = 13)	**9**	**69.2**	**5.2**	**2**	**15.4**	**1.5**	**2**	**15.4**	**2.0**	0.3

* *Global p-value for comparison of each disease between each group. Ab, antibody. Results are presented as the number of patients in each category (percent of subjects in the antibodies group; percent of subjects with the clinical manifestation). For example, 21 subjects with systemic lupus were Ro52+Ro60- which represents 12.2% (21/172) of the Ro52+Ro60- patients and 17.2% (21/122) of the patients with systemic lupus. “Other” diseases were non-auto-immune, non-malignant and non-infectious diseases*.

*Patients “not classified” were patients for whom no diagnosis was established; either because of missing data or because the referring physician did not conclude at last follow-up. Qualitative data were compared with chi square test or, when not possible, the Fischer exact test*.

*The bold values in the table represent the main diseases group*.

**Table 2:** Comparison of patients' indirect immunofluorescence findings, in the Ro52+Ro60-, Ro52+Ro60+ and Ro52-Ro60+ groups.

	**Ro52+Ro60- (*n* = 172)**	**Ro52+Ro60+ (*n* = 130)**	**Ro52-Ro60+ (*n* = 97)**	p^*^
**ANA (median dilution)**	**1/200**	**1/1600**	**1/400**	**<10**^**−4**^
**Nuclear Fluorescence**				**0.03**
Speckled	80 (46.5%)	67 (51.5%)	41 (42.3%)	
Granular Speckled	10 (5.8%)	20 (15.4%)	8 (8.3%)	
Nucleolar speckled	41 (23.8%)	24 (18.5%)	21 (21.7%)	
Other pattern	41 (23.8%)	19 (14.6%)	27 (27.8%)	
**Cytoplasmic Fluorescence**				**0.05**
Negative	67 (39.0%)	49 (37.7%)	54 (55.7%)	
Weakly positive	43 (25.0%)	28 (21.5%)	17 (17.5%)	
Speckled or fine speckled	17 (9.9%)	18 (13.9%)	8 (8.3%)	
Granular	37 (21.5%)	34 (26.2%)	16 (16.5%)	
Other pattern	8 (4.7%)	1 (0.8%)	2 (2.1%)	

* *Global p-value. Results are presented as the number of patients in each category, with their percentages, unless otherwise specified*.

*Qualitative data were compared with chi square test or, when not possible, the Fischer exact test*.

*The bold values in the table represent the significant ones*.

In the Ro52+Ro60+ group, systemic lupus was frequent (41.5%), as was primary Sjögren's syndrome (39.2%). Other diagnoses were far less represented (Table 1). Median ANA fluorescence titer was the highest in this group, at 1/1600 (Table 2). The most frequently associated autoantibodies were anti-La antibodies (36.2%), anti-dsDNA antibodies (54.0%) and RF (55.7%) (Table 3).

**Table 3:** Comparison of patients' anti-ENA antibodies findings, in the Ro52+Ro60-, Ro52+Ro60+ and Ro52-Ro60+ groups.

	**Ro52+Ro60- (*n* = 172)**	**Ro52+Ro60+ (*n* = 130)**	**Ro52-Ro60+ (*n* = 97)**	p^*^
**ENA (*****n*** **= 399)**				
La	2 (1.2%)	47 (36.2%)	4 (4.1%)	**<10**^**−4**^
Ribosome	2 (1.2%)	3 (2.3%)	2 (2.1%)	0.7
CENP-B	8 (4.7%)	4 (3.1%)	2 (2.1%)	0.5
Jo1	7 (4.1%)	1 (0.8%)	2 (2.1%)	0.2
PmScl	4 (2.3%)	1 (0.8%)	1 (1.0%)	0.5
SmRNP	6 (3.5%)	6 (4.6%)	3 (3.1%)	0.8
U1RNP	4 (2.3%)	6 (4.6%)	6 (6.2%)	0.3
HIS	4 (2.3%)	3 (2.3%)	1 (1.0%)	0.7
Sm	2 (1.2%)	2 (1.5%)	1 (1.0%)	0.9
Scl70	1 (0.6%)	0	2 (2.1%)	0.2
Anti-dsDNA (*n* = 331)	40/131 (30.5%)	61/113 (54.0%)	38/87 (43.7%)	**0.001**
RF (*n* = 313)	44/134 (32.8%)	59/106 (55.7%)	21/73 (28.8%)	**0.0002**
Anti-CCP (*n* = 291)	13/123 (10.6%)	7/98 (7.1%)	6/70 (8.6%)	0.7
Anti-TPO (*n* = 265)	7/110 (6.4%)	9/90 (10.0%)	5/65 (7.7%)	0.6
Anti-TG (*n* = 264)	8/110 (7.3%)	9/89 (10.1%)	3/65 (4.6%)	0.4
LAC (*n* = 260)	5/101 (5.0%)	12/96 (12.5%)	10/63 (15.9%)	**0.05**
Anti-B2GP1 (*n* = 291)	3/113 (2.7%)	4/105 (3.8%)	4/73 (5.5%)	0.6
Anti-Cardiolipin (*n* = 292)	9/113 (8.0%)	8/106 (7.6%)	13/73 (17.8%)	**0.05**
Cryoglobulin (*n* = 266)	22/109 (20.2%)	19/87 (21.8%)	16/70 (22.9%)	0.9

* *Global p-value. Results are presented as the number of patients in each category, out of the number of tested patients, with their percentages*.

*Qualitative data were compared with chi square test or, when not possible, the Fischer exact test. ENA, Antibodies against the panel “Extractable Nuclear Antigens”, RF, Rhumatoid Factor; Anti-TPO, anti-thyroperoxydase; anti-TG, anti-thyroglobuline; LAC, Lupus Anti-Coagulant. The bold values in the table represent the significant ones*.

In the Ro52-Ro60+ group, systemic lupus was the most frequent diagnosis (48.5%), whereas only 13.4% of the patients had primary Sjögren's syndrome (Table 1). ANA median fluorescence titer was 1/400 (Table 2) and anti-dsDNA antibodies was the most frequently associated autoantibody (43.7%). Lupus anticoagulant (15.9%) and anti-cardiolipin antibodies (17.8%) were more represented in this group (Table 3).

Finally, in the Ro52+Ro60- group, AID was also the most represented group of disease, even if patients displayed a greater variety of pathologies such as infectious, neoplastic, pulmonary (chronic obstructive pulmonary diseases…) or cardiovascular diseases (myocardial infarction, strokes…). (represented in the “other” diagnosis group) (Table 1). ANA median fluorescence titer was the lowest at 1/200, but cytoplasmic fluorescence was more frequently observed (essentially weakly positive or granular fluorescence) than in the Ro52-Ro60+ group (*p* = 0.05) (Table 2). Association with other autoantibodies was less frequent than in the other two groups (Table 3).

When comparing the 3 different groups, striking differences appeared, concerning associations with AID and other autoantibodies.

Primary Sjögren's syndrome in the Ro52+Ro60+ group was, respectively, four and ten times more prevalent than in the Ro52-Ro60+ and Ro52+Ro60 group (*p* < 10^−4^) (Table 4). Interestingly, patients with the “triad” Ro52, Ro60 and La Ab were even more likely to have Sjögren's syndrome than the double positive patients (53.2% vs. 31.3%, *p* = 0.01). Patients in this group (Ro52+Ro60+) were also more than ten times more likely to have an anti-La antibody than patients with isolated Ro60 Ab (*p* < 10^−4^) and fifty times more likely than patients with isolated Ro52 Ab (*p* < 10^−4^) (Table 5).

**Table 4:** Associations between antibodies profile group and auto-immune diseases (univariate analyses).

	**Ro52+Ro60- vs. Ro52+Ro60+**	**Ro52+Ro60- vs. Ro52-Ro60+**	**Ro52+Ro60+ vs. Ro52-Ro60+**
**OR [CI 95%]**, ***p***			
Systemic lupus	**0.2 [0.1–0.3], < 10**^**−4**^	**0.1 [0.08–0.3], < 10**^**−4**^	0.8 [0.4–1.3], 0.3
Primary Sjögren syndrome	**0.1 [0.06–0.2], < 10**^**−4**^	0.5 [0.2–1.1], 0.09	**4.2 [2.1–8.3], < 10**^**−4**^
Systemic Sclerosis	1.8 [0.5–7.1], 0.4	2.0 [0.4–9.9], 0.3	1.1 [0.2–6.8], 0.9
Inflammatory myositis	**10.5 [1.4–81.7], 0.02**	1.9 [0.6–6.0], 0.3	0.2 [0.0–1.6], 0.1
Inflammatory rheumatism	**4.6 [1.6–13.8] 0.006**	1.3 [0.6–2.8], 0.5	**0.3 [0.1–0.9], 0.03**

*Results are presented as odds ratio with their 95% confidence interval, p-value. Qualitative data were compared with Fisher exact test. The bold values in the table represent the significant ones*.

**Table 5:** Associations between antibodies profile group and other autoantibodies (univariate analyses).

	**OR [CI 95%], p**		
	**Ro52+Ro60- vs. Ro52+Ro60+**	**Ro52+Ro60- vs. Ro52-Ro60+**	**Ro52+Ro60+ vs. Ro52-Ro60+**
**AUTO-ANTIBODIES**			
**ENA**			
La	**0.02 [0.005–0.09], < 10**^**−4**^	0.3 [0.05–1.5], 0.1	**13.2 [4.5–38.1], < 10**^**−4**^
Ribosome	0.5 [0.08–3.0], 0.5	0.6 [0.08–4.0], 0.5	1.1[0.2–6.8], 0.9
CENP-B	1.5 [0.5–5.2], 0.5	2.3 [0.5–11.1], 0.3	1.5 [0.3–8.4], 0.6
Jo1	5.5 [0.7–45.0], 0.1	2.0 [0.4–9.9], 0.4	0.4 [0.03–4.1], 0.4
PmScl	3.1 [0.3–27.8], 0.3	2.3 [0.3–20.7], 0.5	0.7 [0.05–12.0], 0.8
SmRNP	0.7 [0.2–2.4], 0.6	1.1 [0.3–4.6], 0.9	1.5 [0.4–6.2], 0.6
U1RNP	0.5 [0.1–1.8], 0.3	0.4 [0.1–1.3], 0.1	0.7 [0.2–2.3], 0.6
HIS	1.0 [0.2–4.6], 1.0	2.3 [0.3–20.9], 0.5	2.3 [0.2–22.1], 0.5
Sm	0.8 [0.1–5.4], 0.8	1.1 [0.1–12.7], 0.9	1.5 [0.1–13.8], 0.7
Scl70	*NI*	0.3 [0.03–3.1], 0.3	*NI*
Anti-dsDNA	**0.4 [0.2–0.6], < 10**^**−4**^	**0.6 [0.3–0.996], 0.048**	1.5 [0.9–2.7], 0.1
RF	**0.4 [0.2–0.7], < 10**^**−4**^	1.2 [0.7–2.3], 0.5	**3.1 [1.6–5.9], < 10**^**−4**^
Anti-CCP	1.5 [0.6–4.0], 0.4	1.3 [0.5–3.5], 0.7	0.8 [0.3–2.6], 0.7
Anti-TPO	0.6 [0.2–1.7], 0.3	0.8 [0.2–2.7], 0.7	1.3 [0.4–4.2], 0.6
Anti-TG	0.7 [0.3–1.9], 0.5	1.6 [0.4–6.3], 0.5	2.3 [0.6–9.0], 0.2
LAC	**0.4 [0.1–1.1], 0.07**	**0.3 [0.1–0.9], 0.02**	0.8 [0.3–1.9], 0.5
Anti-B2GP1	0.7 [0.2–3.2], 0.6	0.5 [0.1–2.2], 0.3	0.7 [0.2–2.8], 0.6
Anti-Cardiolipin	1.1 [0.4–2.9], 0.9	**0.4 [0.2–0.99], 0.047**	**0.4 [0.1–0.96], 0.04**
Cryoglobulin	0.9 [0.5–1.8], 0.8	0.9 [0.4–1.8],0.7	0.9 [0.4–2.0], 0.9

*Results are presented as odds ratio with their 95% confidence interval, p-value. Qualitative data were compared with Fisher exact test. NI, Non-Interpretable, as n = 0 in the Ro52+Ro60+ group. ENA, Extractable Nuclear Antigens, tested by FIDIS™; RF, Rhumatoid Factor; Anti-TPO, anti-thyroperoxydase; anti-TG, anti-thyroglobuline, LAC, Lupus Anti-Coagulant. The bold values in the table represent the significant ones*.

In systemic lupus was equally prevalent in both Ro52-Ro60+ and Ro52+Ro60+ groups (*p* = 0.3) (Tables 1, 4). It is noteworthy that Ro52-Ro60+ patients showed a slightly higher prevalence of anti-cardiolipin antibodies (*p* = 0.05) and lupus anticoagulant (*p* = 0.02) (Table 5), but not anti β2GPI antibodies (*p* = 0.6) (Table 3).

Inflammatory myositis was ten times more likely in the Ro52+Ro60- group than in the Ro52+Ro60+ group (*p* = 0.02) (Table 4).

Inflammatory rheumatism was also more frequently observed in this group compared with the double positive group (*p* = 0.006) (Table 4).

Interestingly, out of 12 patients with systemic sclerosis, seven displayed isolated Ro52 Ab (*p* = 0.6) (Table 1).

There was no specific association with other auto-antibodies in this group of patients (Table 5).

Concerning specific disease presentation, differences were also observed between the 3 groups.

In systemic lupus, patients in the Ro52+Ro60+ group were more likely to have cytopenias (*p* = 0.04) and hypergammaglobulinemia (*p* = 0.01), whereas patients in the Ro52+Ro60- group were more at risk for renal insufficiency (*p* = 0.04) and needed more lines of treatment than patients in the other groups (*p* = 0.02). However, there was no significant difference in vital status (Table 6).

**Table 6:** Disease severity in systemic lupus, Sjögren's syndrome, systemic sclerosis and inflammatory myositis depending on antibodies profile group (univariate analysis).

		**Ro52+Ro60-**	**Ro52+Ro60+**	**Ro52-Ro60+**	p^*^
Systemic lupus (*n* = 122)		***n*** **= 21**	***n*** **= 54**	***n*** **= 47**	
	Alive (%)	21 (100.0)	52 (96.3)	47 (100.0)	0.4
	Anemia (%)	3/20 (15.0)	**12/53 (22.6)**	8/45 (17.8)	**0.04**
	Thrombopenia (%)	1/20 (5.0)	**10/53 (18.9)**	5/45 (11.1)	**0.02**
	Lymphopenia (%)	5/20 (25.0)	**16/51 (31.4)**	7/45 (15.6)	**0.008**
	Hypergammaglobulinemia (%)	**3/8 (37.5)**	**12/29 (41.4)**	3/19 (15.8)	**0.01**
	Renal insufficiency (%)	**4/19 (21.1)**	7/53 (13.2)	7/44 (15.9)	**0.04**
	Lines of treatment (median [IQR])	**3.0 [1.0-4.0]**	2.0 [1.0-3.0]	2.0 [2.0-4.0]	**0.02**
Sjögren's syndrome (*n* = 76)		***n*** **= 12**	***n*** **= 51**	***n*** **= 13**	
	Alive (%)	91.7	96.0	100.0	0.2
	Anemia (%)	**3/12 (25.0)**	7/50 (14.0)	0/12	**0.03**
	Thrombopenia (%)	1/12 (8.3)	2/50 (4.0)	1/12 (8.3)	0.1
	Lymphopenia (%)	2/12 (16.7)	9/48 (18.8)	1/11 (9.1)	0.09
	Hypergammaglobulinemia (%)	0/9	**16/42 (38.1)**	0/7	**0.002**
	Renal insufficiency (%)	**5/12 (41.7)**	8/47 (17.0)	1/12 (8.3)	**0.01**
	Lines of treatment (median [IQR])	2.0 [0.0-2.0]	2.0 [1.0-2.0]	1.0 [1.0-2.0]	0.3
Systemic sclerosis (*n* = 12)		***n*** **= 7**	***n*** **= 3**	***n*** **= 2**	
	Alive (%)	6/7 (85.7)	2/3 (66.7)	**0/2**	**0.04**
	Anemia (%)	3/7 (42.9)	1/3 (33.3)	1/2 (50.0)	0.3
	Thrombopenia (%)	1/7 (14.3)	0/3	0/2	0.6
	Lymphopenia (%)	2/7 (28.6)	0/3	0/2	0.3
	Hypergammaglobulinemia (%)	1/5 (20.0)	1/2 (50.0)	1/1 (100.0)	0.2
	Renal insufficiency (%)	2/7 (28.6)	1/3 (33.3)	0/1	0.4
	Lines of treatment (median [IQR])	1.0 [0.0-3.0]	1.0 [1.0-2.0]	3.0 [1.0-5.0]	0.6
	Diffuse skin involvement (%)	0/7	0/3	**2/2 (100.0)**	**0.02**
	Pulmonary hypertension (%)	1/7 (14.3)	0/3	1/2 (50.0)	0.2
	Pulmonary fibrosis (%)	3/6 (50.0)	1/3 (33.3)	2/2 (100.0)	0.1
Inflammatory myositis (*n* = 18)		***n*** **= 13**	***n*** **= 1**	***n*** **= 4**	
	Alive (%)	13/13 (100.0)	1/1 (100.0)	4/4 (100.0)	na
	Anemia (%)	2/13 (15.4)	1/1 (100.0)	0/4	0.09
	Thrombopenia (%)	0/13	0/1	0/4	na
	Lymphopenia (%)	2/13 (15.4)	0/1	0/4	0.5
	Hypergammaglobulinemia (%)	1/10 (10.0)	1/1 (100.0)	0/2	0.1
	Renal insufficiency (%)	1/12 (8.3)	0/1	0/4	0.7
	Lines of treatment (median [IQR])	3.0 [2.0-.05]	2.0 [2.0-2.0]	1.5 [0.5-2.5]	0.2

* *Global p-value. Qualitative data are presented as number of patients in each antibodies profile group with their percentages (out of the number of tested patients for each feature), and were compared with chi square test or, when not possible, the Fischer exact test. Quantitative data are presented as a median with their interquartile range ([IQR]) and were compared with Mann-Whitney test. Alive: percentage of patients still alive at last follow-up. NA, not applicable. The bold values in the table represent the significant ones*.

In Sjögren's syndrome, patients in the Ro52+Ro60+ group were more likely to have hypergammaglobulinemia (*p* = 0.002). Patients in the Ro52+Ro60- group were more at risk for anemia (*p* = 0.03) and renal insufficiency (*p* = 0.01). We did not observe differences in vital status nor in number of lines of treatment (Table 6).

In systemic sclerosis, the only two patients with diffuse skin involvement were in the Ro52-Ro60+ group, and were the only patients with systemic sclerosis who died during follow-up. We did not observe significant differences in heart or lung involvement (Table 6).

There were no clinical differences in inflammatory myositis presentation (Table 6).

## Discussion

The diagnostic utility of the individual detection of Ro52 and Ro60 Ab is still disputed. Indeed, some immunology laboratories in France have even stopped providing Ro52 Ab findings. This can be confusing for clinicians who are not necessarily aware of what is tested when asking for anti-SS-A antibodies.

We report here a new study, including 399 patients, that distinguishes three distinct phenotypes depending on Ro52 Ab and/or Ro60 Ab presence or absence (Ro52+Ro60-, Ro52+Ro60+, Ro52-Ro60+) in a selected hospital-population. Patients with both Ro52 and Ro60 Ab were far more likely to be diagnosed with primary Sjögren's syndrome or in a less remarkable fashion, systemic lupus. Patients with isolated Ro60 Ab were more frequently diagnosed with systemic lupus and might have more lupus anti-coagulant and anti-cardiolipin antibodies. Patients with isolated Ro52 Ab presented with more varied pathologies but among AID, inflammatory myositis and inflammatory rheumatism were far more represented.

Ro52 Ab were the most frequent antibodies in this hospital population. Similarly, Murng et al. recently reported that Ro52 Ab were relatively common in a similar population (29). Ro52 Ab might be more prevalent in hospital-patients than Ro60 Ab, thus explaining the wider variety of diseases found in these patients. In the general population, only a few studies have evaluated Ro Ab prevalence (30) and only one of these concerned Ro52 Ab. Indeed, Menendez et al. examined 50 sera from blood donors and none had Ro52 Ab (15). In the same fashion, none of the healthy donors tested in our study had Ro52 Ab.

We also confirm here that isolated Ro52 Ab have a different biological and clinical significance.

Biologically, cytoplasmic fluorescence was more frequent in patients with isolated Ro52 Ab, as found by other teams (31, 32). This could be due to the fact that TRIM21 steady-state subcellular localization is cytoplasmic and becomes nuclear only under pro-inflammatory stimuli (interferon α) (4, 33, 34). In some cases, this cytoplasmic fluorescence could also be due to the presence of anti-synthetase antibodies. Indeed, these antibodies (anti-JO1,anti-PL7 or PL12 antibodies) are associated with dense fine speckled or fine speckled cytoplasmic fluorescence on Hep2 cells (35).

Clinically, AID were less represented in patients with isolated Ro52 Ab compared with patients in the other groups. This is in accordance with other studies where AID represented only 31.3 to 73.5% of the diagnosis (15, 17). These patients also had more infectious and malignant diseases as previously described (11, 14). These differences in the repartition of diagnoses might explain the prevalence of older-aged patients and lower female/male *sex ratio* than in the other two groups.

In an autoimmune context, isolated Ro52 Ab may be of particular interest in the diagnosis of inflammatory myositis, as suggested by other observations (12, 15, 16, 31, 36, 37). This association is particularly interesting as patients with inflammatory myositis and Ro52 Ab may present with a more severe disease and poorer response to usual immunosuppressive treatment but better response to rituximab (38).

As reported by other teams, we also observed that patients with systemic sclerosis seemed to be more likely to have isolated Ro52 Ab (39, 40). Separate detection of Ro52 Ab could be of particular importance in systemic sclerosis, as it could be associated with interstitial lung disease and a more severe disease (19, 41).

In the Ro52+Ro60+ group, primary Sjögren's syndrome was the most frequent diagnosis. This association was particularly striking when anti-La antibodies were also positive. Association with RF was also particularly frequent. These associations are not surprising since RF, even if not a specific marker, and anti-La antibodies are known to be associated with Sjögren's syndrome (42–44). Similar data are found in literature as shown in Table 7 and as supported by Popovic et al. (patients with primary Sjögren's syndrome had higher titer of Ro52 Ab and anti-La antibodies than patients with cutaneous lupus or systemic lupus) (45).

**Table 7:** Comparative literature review.

			**Antibodies profile**			
	**Study design**	**Diseases**	**Ro52+Ro60-**	**Ro52-Ro60+**	**Ro52+Ro60+**	**Ro52+Ro60+La+**
Dugar et al. (16)	Sera from patients with different AID	Sjögren'syndrome (*n* = 40)	4/40 (10.0%)	0	10/40 (25.0%)	24/40 (60.0%)
		Systemic lupus (*n* = 67)	4/67 (5.9%)	11/67 (16.5%)	22/67 (32.8%)	12/67 (17.9%)
		Systemic sclerosis (*n* = 106)	10/106 (9.4%)	0	3/106 (2.8%)	2/106 (1.9%)
		Inflammatory myositis (*n* = 147)	23/147 (15.6%)	0	12/147 (8.2%)	0
Gonzalez et al. (32)	Sera tested for anti-Ro-52 and anti-Ro60 antibodies	Sjögren'syndrome (*n* = 48)	8/48 (16.7%)	2/48 (4.2%)	6/48 (12.5%)	27/48 (56.2%)
		Systemic lupus (*n* = 33)	6/33 (18.2%)	6/33 (18.2%)	4/33 (12.1%)	9/33 (27.3%)
		Systemic sclerosis	NA	NA	NA	NA
		Inflammatory myositis (*n* = 2)	0	1/2 (50%)	0	1/2 (50%)
Bahon et al. (18)	Sera tested positive for anti-Ro52 and/or Ro60 and/or La antibodies	Sjögren'syndrome (*n* = 48)	14/48 (29.2%)	1/48 (2.1%)	9/48 (18.8%)	21/48 (43.8%)
		Systemic lupus (*n* = 57)	7/57 (12.3%)	10/57 (17.5%)	19/57 (33.3%)	13/57 (22.8%)
		Systemic sclerosis (*n* = 4)	0	NA	NA	NA
		Inflammatory myositis (*n* = 6)	4 /6 (66.7%)	NA	NA	NA
Menendez et al. (15)	Sera tested positive for anti-Ro52 and/or Ro60 antibodies	Sjögren'syndrome (*n* = 35)	7/35 (20.0%)	3/35 (8.6%)	25/35 (71.4%)	NA
		Systemic lupus (*n* = 47)	4/47 (8.5%)	9/47 (19.2%)	34/47 (72.3%)	NA
		Systemic sclerosis (*n* = 6)	4/6 (66.7%)	0	2/6 (33.3%)	NA
		Inflammatory myositis (*n* = 6)	6/6 (100%)	0	0	NA
Murng et al. (29)	Sera tested positive for anti-Ro52 antibodies	Sjögren'syndrome (*n* = 44)	10/44 (22.7%)	NA	34/44 (77.3%)	NA
		Systemic lupus (*n* = 31)	8/31 (25.8%)	NA	23 (74.2%)	NA
		Systemic sclerosis (*n* = 3)	3/3 (100%)	NA	0	NA
		Inflammatory myositis (*n* = 2)	2/2 (100%)	NA	0	NA

*Comparison of results from similar studies (based on sera positive for anti-Ro52 and/or Ro60 antibodies), for the main auto-immune diseases (Sjögren's syndrome, systemic lupus, systemic sclerosis and inflammatory myositis). NA, not applicable because of study design or not enough data in publication*.

Ro52 Ab (isolated or not) may also be related to disease severity.

Even if our study was not designed to evaluate differences in severity for each AID, we highlighted some differences in clinical presentation. Indeed, patients with systemic lupus and isolated Ro52 Ab seemed to have more severe symptoms, with more renal involvement and necessity of a higher number of lines of treatment, whereas patients with Ro52 and Ro60 Ab were more susceptible to hematologic complications as found by Menendez et al. (46) Patients with Sjögren's syndrome and Ro52 Ab also seemed to have more severe symptoms with more renal insufficiency and anemia as found by Retamozo et al. (patients with Sjögren's syndrome and high titer of Ro52 Ab had more anemia and leukopenia) (47). In pregnant women, Ro52 Ab are more associated with congenital heart block and their pathogenicity was proven in animal models (13, 22, 48–50).

Finally, isolated Ro60 Ab were associated with systemic lupus, as previously described by some authors (51). It was also associated with lupus anticoagulant and anti-cardiolipin antibodies presence. This association has rarely been described or studied to date in other publication (15, 17), a negative association has been described in one study (46). It might be due to a particular phenotype of patients with systemic lupus and secondary anti-phospholipid syndrome or at least anti-phospholipid antibodies (28).

In short, we feel that Ro52 and Ro60 Ab should be detected separately as (i) isolated Ro52 Ab is more frequently associated with inflammatory myositis and inflammatory rheumatism; (ii) Ro52 Ab presence could help identify different sub-groups of patients at risk in primary Sjögren's syndrome (hematological involvement), in inflammatory myositis (severity, treatment response), in systemic sclerosis (lung involvement) and in pregnant women (fetal heart block); (iii) isolated Ro60 Ab are more likely to be seen in systemic lupus, and might have a particular association with anti-phospholipid antibodies; (iv) double positive patients are far more likely to be diagnosed with primary Sjögren's syndrome than patients with isolated Ro52 or Ro60 Ab.

However, our study has some limitations. Diagnoses were based on the referring physician appreciation and not upon international classification criteria. We chose to do so to be as near as possible to “real life” problematics, since diagnoses in AID are based upon of body of evidence and classification criteria are not diagnostic criteria. Moreover, patients were followed for many years before data collection, ensuring sufficient hindsight upon diagnosis. Even if our results seem coherent with other publication, we should bare this limitation in mind when interpreting our study.

Moreover, our study is based upon a selected population of patients taken care of in a university hospital center (mostly in rheumatology and internal medicine wards). We do not know if the prevalence of Ro52 Ab is similar in the general population. These results should therefore not be extended to the general population. A prospective study, including a larger control group, should be performed to validate these findings.

We therefore suggest that, when anti-ENA antibodies are prescribed in a suspected auto-immune context, it should include separate Ro52 and Ro60 Ab determination. To go even further, we would like to suggest a change in ENA nomenclature, to avoid any confusion, abandoning the “anti-SS-A” label in favor of the “anti-TRIM21” or “anti-Ro60” antibodies designation.

## Ethics Statement

This study was carried out in accordance with the declaration of Helsinki. Ethical approval from an institutional review board was not necessary since no other data were collected than those already in the patient's medical record (Hospital agreement with the French data protection authority: CNIL).

## Author Contributions

AR, DG, and B-NP participated in the design of the study. AR and ST assured the data collection. MH carried out all the statistical analyses. AR, KD, DG, B-NP, and AS prepared the manuscript. All the authors made critical revision of the manuscript. All the authors read and approved the final manuscript.

### Conflict of Interest Statement

The authors declare that the research was conducted in the absence of any commercial or financial relationships that could be construed as a potential conflict of interest.
